# Ultrafast NH and CH Vibrational Dynamics in Hydrogen-Bonded 2‑Pyrrolidinone

**DOI:** 10.1021/acsomega.5c08358

**Published:** 2025-09-16

**Authors:** Kiran Sankar Maiti

**Affiliations:** TUM School of Natural Sciences, Department of Chemistry, 9184 Technical University of Munich, 85748 Garching, Germany

## Abstract

Over the past two and a half decades, two-dimensional infrared (2DIR) spectroscopy has undergone significant methodological and technological advancements, enabling the exploration of numerous physical and chemical processes in complex molecules, particularly biological ones. Among these, interactions between vibrational modes, such as NH and CH stretches, are of special interest in biological contexts. However, these interactions have not been thoroughly investigated until now. In this study, broadband 2DIR spectroscopy, spanning a spectral range of 2750 cm^–1^ to 3350 cm^–1^, is employed to examine the molecular structure and dynamics of 2-pyrrolidinone. This approach provides time-resolved insights into the coupling of NH and CH stretch vibrations. Distinct signatures of chemical exchange, as well as coherent and incoherent couplings, are observed. The chemical exchange arises from the pseudorotation of the five-membered ring, transitioning between axial and equatorial conformers. Coherent coupling is identified between symmetric and asymmetric CH stretch vibrations, while incoherent coupling is observed between asymmetric CH stretch and NH stretch vibrations. Furthermore, intermolecular hydrogen bonding in 2-pyrrolidinone enables the detailed study of hydrogen bond making and breaking processes, which are fundamental to the behavior of biological molecules. This investigation sheds light on the intricate vibrational dynamics and interactions in 2-pyrrolidinone, offering valuable insights into processes critical to biological systems.

## Graphical abstract

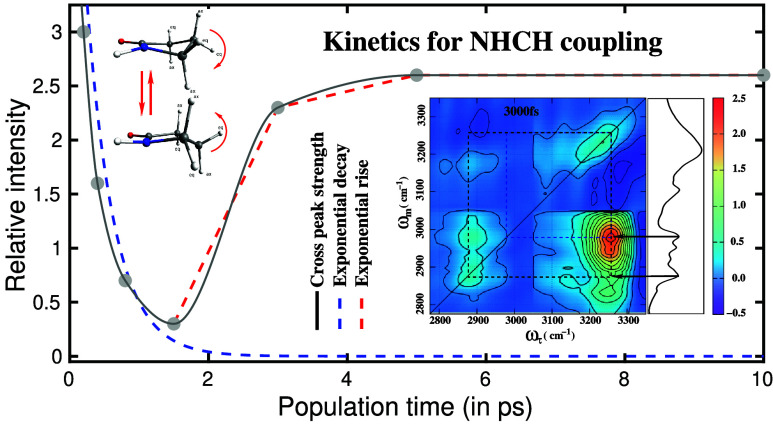

## Introduction

Many critical chemical and biological processes are initiated by rapid molecular conformational changes in the condensed phase.^1–4^ In this environment, molecular vibrational frequencies are influenced not only by the bond connectivity within molecules but also by interactions between solutes and solvents.^5,6^ These local and nonlocal interactions significantly affect the vibrational bands of molecules, complicating efforts to understand their structure and dynamic behavior.^7^ However, understanding molecular structure and their dynamics are crucial for unraveling the biological processes they govern.^8–11^ Over the past few decades, considerable effort has been dedicated to developing tools capable of monitoring three-dimensional molecular conformations in real time. Among these methods, two-dimensional infrared spectroscopy (2DIR) stands out as a highly promising technique.^12,13^ With its essentially unlimited time resolution for large molecular motions, 2DIR provides a powerful means to visualize structural changes in complex molecules.^14–18^

In vibrational spectroscopy, CH and NH stretching vibrations are essential for determining the structure of biological molecules.^19–22^ It is believed that CH stretch vibrations, occurring in the range of 2800 cm^–1^ to 3100 cm^–1^, are mostly unaffected by interference from other chemical groups in biological molecules. In reality, CH bands actively interact with intramolecular bands and play a crucial role in determining molecular structure.^23,24^ However, their weak infrared response compared to other vibrational bands makes them challenging to identify any interaction to other chemical groups.^25–27^ On the other hand, the NH stretching vibrational band is relatively isolated and exhibits a strong infrared response. Due to nitrogen’s high electron affinity, the NH vibrational band acts as a potent hydrogen donor, facilitating intra- and intermolecular hydrogen bonding.^5,28^ Hydrogen bonding significantly affects the vibrational spectra of molecules, causing the NH vibrational band to undergo notable redshifts and broadening across a wide spectral range depending on the bond strength.^29,30^ The NH vibrational band not only participates in hydrogen bonding but also significantly influences the vibrations of other bands and exhibits strong coupling.^30^ The NH vibrational band in proteins and peptides has been extensively studied.^5,14^ However, to the best of current knowledge, the coupling between CH and NH vibrational bands has not been explored. Two-dimensional infrared (2DIR) spectroscopy has demonstrated its effectiveness in disentangling such vibrational couplings with high efficiency.^26,31^

Over the past two and a half decades, significant technical and theoretical advancements have been made in 2DIR spectroscopy.^32–40^ Most of these efforts have focused on analyzing one or a few closely spaced vibrational bands of the molecule, which limits the amount of structural information that can be obtained. Due to the significant technical challenges involved, this technique is still not widely used to investigate the complete molecular picture.^41^ Recently, however, several attempts have been made to expand the accessible frequency range using multimode 2DIR spectroscopy.^42,43^

In this study, a straightforward experimental approach was employed to explore the vibrational dynamics and coupling between CH and NH vibrational bands in biomolecules. The experiment utilizes a collinear pump pulse pair to excite the molecule, while a noncollinear laser pulse serves as a probe to collect the absorptive spectra.^44^ 2-pyrrolidinone was chosen as a model biomolecule because it exhibits many characteristic features of proteins and peptides. Its hydrogen-bonding properties make it particularly suitable for studying structural sensitivity, a key attribute of biomolecules.^45^ Additionally, the conformational changes of 2-pyrrolidinone enabled the investigation of chemical exchange processes, which are the central focus of this article. The structure and conformations of 2-pyrrolidinone are shown in Figure 1. Additionally, its moderate size allows for reasonably accurate ab initio calculations.^45,46^ The C=O double bond in its structure serves as a model system for the amide-I band of protein backbones.

**Figure 1:**
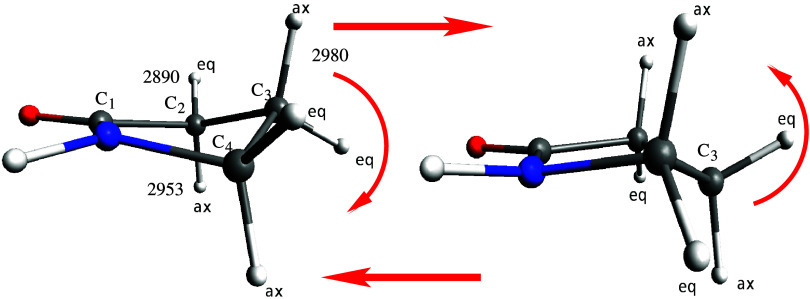
Molecular conformers and pseudorotation of 2-pyrrolidinone from equatorial and axial configuration.

## Experimental Procedures

An ultrafast regenerative, multipass Ti:sapphire amplifier (Integra-C, Quantronix) is used to generate initial femtosecond pulses at 800 nm. The amplifier produces transform-limited pulses of 100 fs duration with 2.5 mJ energy at a 1 kHz repetition rate. The output beam is split into two parts with a 40:60 ratio, both of which are used to pump two optical parametric amplifiers (OPAs; TOPAS, Light Conversion). In combination with a noncollinear difference frequency generator (NDFG, Light Conversion), the TOPAS operating at 60% input power generates a tunable pump beam (ranging from 2750 to 3400 cm^–1^), while another TOPAS independently produces a tunable probe beam within the same spectral range. This setup enables two-color experiments over a broad spectral range.

In a two-dimensional photon echo experiment with a pump–probe geometry, the pump beam is split into two separate beams to generate two pump pulses. These pulses are directed onto retroreflectors mounted on independently computer-controlled translation stages, enabling precise control of the time delay between them. The two pump pulses are then recombined collinearly using a zinc selenide (ZnSe) beam splitter and focused onto the sample at the same point by an off-axis parabolic mirror. The probe pulse is also focused onto the sample by the same parabolic mirror and intersects the pump pulses at a small angle (approximately 12°) within the sample. Further details of the experimental setup can be found in other sources.^35^

To generate and detect the echo signal, one of the pump pulses is modulated using an optical chopper operating at 500 Hz. Within the sample, the interaction of three excitation pulses produces a vibrational echo signal, which is emitted in the same direction as the probe beam. The probe beam functions as a local oscillator (LO) for self-heterodyne detection of the echo signal. The echo is detected using spectral interferometry, providing the signal as a function of the detected frequency dimension, ω_*m*_. The coherence time, τ, is varied while keeping the population time, *T*_*w*_, constant. The signal is then Fourier-transformed along the τ dimension to obtain the excitation frequency dimension, ω_τ_. The signal is spectrally resolved using a Horiba Jobin Yvon iHR320 spectrometer and detected by a 64-element MCT double array (Infrared Systems Development).

The sample, 2-Pyrrolidinone (C_4_H_7_NO), was purchased from Sigma-Aldrich with 99.9% purity and dissolved in CCl_4_ (99.9%) without further purification. The sample concentration was optimized to 20% by volume to ensure sufficient signal strength for the experiment at CH–NH stretch vibrational spectral region. At this sample concentration, a wide range of hydrogen-bonded molecular species forms in the solution, enabling a detailed study of hydrogen-bonding characteristics. The hydrogen-bonded species are illustrated in Figure 2. A custom-built sample cell, comprising two 2 mm thick CaF2 windows separated by a 15 μm Teflon spacer, was used to contain the sample during photon echo measurements. All experiments were conducted at room temperature (21 °C), and the entire system was purged with dry air to eliminate water vapor.^47^

**Figure 2:**
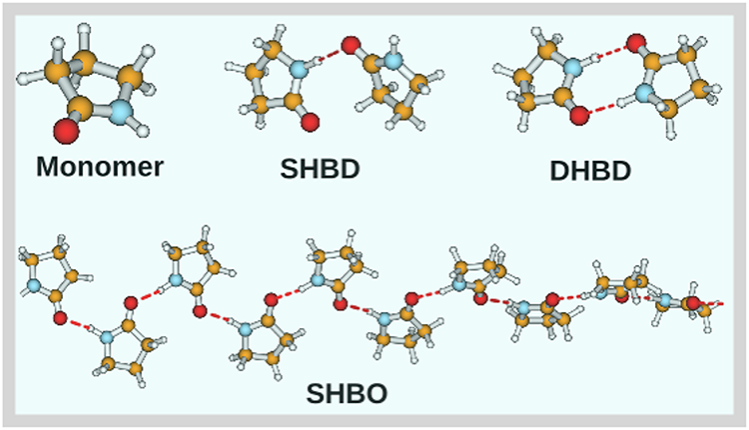
Structure of 2-Pyrrolidinone molecule. The dashed line defines the intermolecular hydrogen bonding. The single molecule labeled as “Monomer”, “SHBD” stand for singly hydrogen bonded dimer, “DHBD” stand for doubly hydrogen bonded dimer, and single hydrogen bonded oligomer define as SHBO.

## Results and Discussion

This study presents two-color, two-dimensional vibrational echo spectra of 2-pyrrolidinone in CCl_4_, recorded at various population times. Each broadband spectrum features four quadrants, representing all combinations of CH and NH stretch vibrational bands. For visual clarity, these quadrants are separated by white dashed lines in Figure 3. The quadrants are labeled according to the pump and probe combinations, with the pump band listed first--for example, “NHCH” refers to excitation of the NH stretch and probing of the CH region. As is typical in 2DIR spectra, both positive and negative peak intensities are observed: red contours denote positive peaks, while blue contours indicate negative ones.

**Figure 3:**
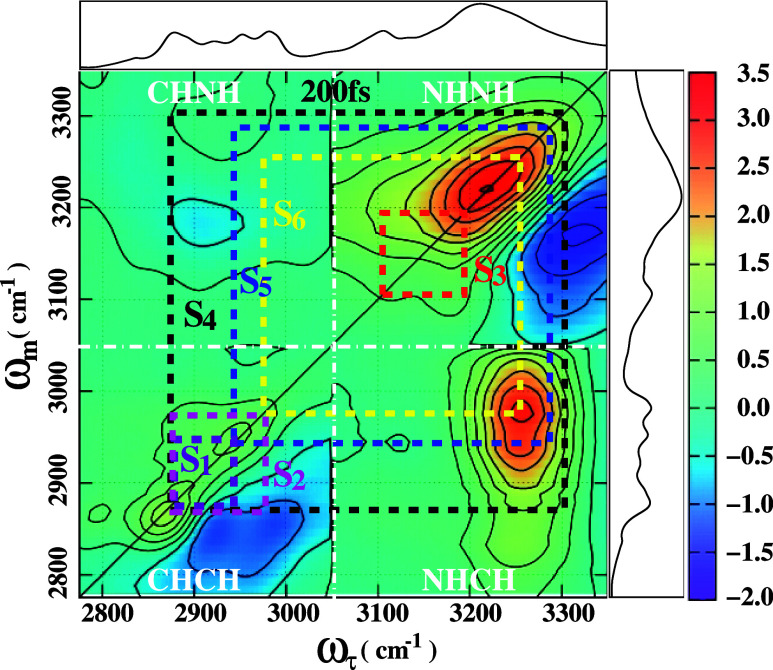
Two dimensional infrared vibrational photon echo broad band real spectrum along with the FTIR spectrum of 2-pyrrolidinone in CCl_4_ at a population tine *T*_*w*_ = 200 fs in room temperature. Two dimensional data have been normalized to the strongest peak. The red contours are positive-trending and the blue contours are negative-trending. The horizontal axis is the Fourier transformed τ-axis (ω_τ_-axis) and the vertical axis is the monochromator axis (ω_*m*_-axis).

Figure 3 displays the broadband two-dimensional vibrational echo spectrum of 2-pyrrolidinone at a population time of *T*_*w*_ = 200 fs. The spectral window covers the CH stretch (∼2800 to 3100 cm^–1^) and the NH stretch (∼3250 cm^–1^) regions.^48^ As the IR pulse duration is approximately 100 fs, determined through three-beam cross-correlation in a nonresonant solvent, nonresonant contributions are not expected at *T*_*w*_ = 200 fs. The spectrum exhibits multiple diagonal and cross peaks, hallmarks of 2D spectroscopy. To aid in peak identification, one-dimensional infrared (1DIR) absorption spectra are placed along the top and right edges of the 2DIR plot. All intensities are normalized to the strongest positive peak at 3250 cm^–1^. The characteristic square pattern^49^ formed by diagonal and cross peaks in 2DIR spectra has been annotated with colored dotted squares to emphasize different coupling types, which are detailed in the subsequent sections.

A broad and intense positive peak appears on the diagonal in the upper-right corner of the spectra in Figure 3. This broad feature arises from ground state bleaching and excited state emission of the hydrogen-bonded NH stretch vibration (ω_τ_ = ω_*m*_ = 3225 cm^–1^).^50^ A detailed analysis of this peak has been previously reported.^29^ For convenience, a brief summary is provided here. A concentration-dependent infrared analysis, supported by ab initio calculations, demonstrated that 2-pyrrolidinone forms a broad distribution of hydrogen-bonded oligomers at the experimental concentration.^45,51^ The strength of these hydrogen bonds increases with oligomer chain length, resulting in a red-shift of the NH vibrational frequency as the bond strength grows.^30,52^ Accordingly, NH vibrational bands from shorter oligomers appear at higher frequencies, while longer chains produce more red-shifted features, contributing to the broad spectral profile. The strongest hydrogen bonding occurs in doubly hydrogen-bonded dimers (DHBDs), which give rise to a relatively weak diagonal peak at ω_τ_ = ω_*m*_ = 3106 cm^–1^ due to the NH stretch vibration. These weak and broadened features are consistent with the corresponding one-dimensional spectra (see Figure 3). Additionally, a broad, intense negative peak is observed in the NH stretch region at ω_τ_ = 3310 cm^–1^ and ω_*m*_ = 3180 cm^–1^, attributed to excited-state absorption of the hydrogen-bonded NH vibration. The elongation of this peak parallel to the diagonal reflects substantial inhomogeneous broadening.

A relatively low-intensity positive diagonal peak is observed at (ω_τ_ = ω_*m*_ = 2890 cm^–1^), corresponding to the ground state bleach and excited state emission of the symmetric CH stretch vibration.^53,54^ This peak extends along the diagonal but is less broadened than the NH vibrational peak. The reason is straightforward: the NH group is directly involved in hydrogen bonding, which strongly influences its vibrational frequency and leads to significant broadening of the NH band. In contrast, the CH group is not directly affected by hydrogen bonding, resulting in only minor changes to its vibrational frequency and less broadening of the CH band. The asymmetric CH stretch vibrational mode, which occurs at slightly higher energy, appears on the diagonal at ω_τ_ = ω_*m*_ = 2953 cm^–1^. Although the asymmetric CH stretch peak is less intense than the symmetric one, it also extends diagonally. These two peaks are identified as equatorial (2890 cm^–1^) and axial (2953 cm^–1^) CH stretch vibrations of the two CH bonds adjacent to the NH bond. A less prominent absorption peak is observed at ω_τ_ = ω_*m*_ = 2980 cm^–1^, arising from the absorption of the CH band adjacent to the C_3_ carbon atom. This specific CH band plays a significant role in the conformational dynamics of the molecule, which will be explained in detail later. A negative peak parallel to the diagonal is observed around (ω_τ_ = 2960 cm^–1^, ω_*m*_ = 2810 cm^–1^), which is attributed to the excited state absorption of the symmetric and asymmetric CH stretch vibration bands. This negative peak is similarly elongated parallel to the diagonal, leading to inhomogeneous broadening.

Apart from the diagonal peaks, the spectrum is rich in numerous cross peaks, the most informative characteristic feature of 2DIR spectroscopy. The positions and strength of these cross peaks provide information about the coupling between participating vibrational bands and their coupling strength, respectively. Generally, the cross peaks form a square pattern in conjunction with their corresponding diagonal peaks, which helps in identifying the origin of the cross peaks. A small square pattern (S_1_) is formed in the lower left corner of the spectrum. A closer examination (see Figure 3) reveals that the equatorial and axial CH stretching modes are coherently coupled, resulting in a positive cross peak at (ω_τ_ = 2890 cm^–1^, ω_*m*_ = 2953 cm^–1^). The coupling between equatorial and axial CH bands occurs naturally because both hydrogen atoms are bonded to the same carbon atom (C_2_), causing the vibration of one to influence the other.^31,55^ A similar positive cross peak is expected at the opposite corner of the square, but it is obscured by a strong negative peak due to the excited-state absorption of the CH stretching band (explained earlier). However, the formation of the corresponding positive cross peak is evidenced by the distortion of the negative peak observed at positions (ω_τ_ = 2953 cm^–1^, ω_*m*_ = 2890 cm^–1^). Furthermore, at longer waiting times, this cross peak becomes more pronounced (see Figure 4), as the negative peak diminishes in intensity. The CH band adjacent to the C_3_ carbon atom appears to couple with the symmetric CH stretch vibration, forming a square pattern (indicated by the dotted square S_2_). This coupling pattern becomes more pronounced at longer waiting times.

**Figure 4:**
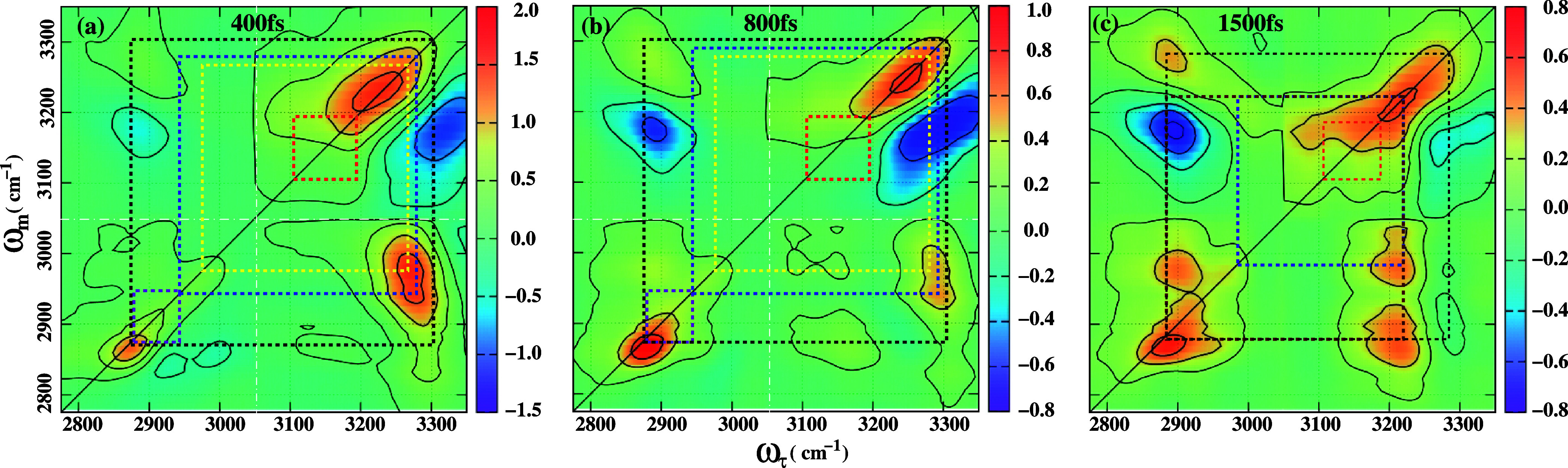
2DIR spectra of 2-pyrrolidinone recorded at different waiting times are presented: (a) *T*_*w*_ = 400 fs, (b) *T*_*w*_ = 800 fs, and (c) *T*_*w*_ = 1500 fs. While all three spectra span the same spectral range, the peak intensities are scaled relative to the highest intensity peak shown in Figure 3.

Several additional coupling patterns are observed between the NH stretching vibration and various CH vibrational bands,^23^ among them some yielding prominent cross peaks. Typically, intensity of cross peaks increase with waiting time.^49^ At early waiting times, cross peaks are practically invisible due to their low intensity and the strong diagonal peaks that obscure them. In relation to the dynamic time scale, 200 fs is too early for strong cross peaks to emerge. Moreover, since all peaks in a broad 2DIR spectrum are normalized to the most intense peak, the cross peaks at 200 fs are not particularly prominent, even though the cross peaks themselves are reasonably intense. Nevertheless, some cross peaks are still visible. For instance, a red square (S_3_) pattern in the NH vibrational region and a yellow dotted square (S_6_) between the NH and CH vibrational regions highlight a few prominent cross peaks, which are discussed in more detail later.

The red square (S_3_) feature observed on the lower frequency side of the broad NH diagonal peak arises due to the NH vibrational coupling between DHBD and SHBO. A comprehensive analysis of this coupling process has been reported earlier; for convenience, a brief summary is presented below.^29^ A relatively strong peak appears at one corner of the square at (ω_τ_ = 3106 cm^–1^, ω_*m*_ = 2995 cm^–1^), while the antithetical cross peak, though less intense, is seen at (ω_τ_ = 2995 cm^–1^, ω_*m*_ = 3106 cm^–1^). This square feature reflects hydrogen bond exchange between DHBD and SHBO. In one process, one of the hydrogen bond from DHBD breaks and forms a new bond with a long-chain oligomer, attaching to it. In the other process, two 2-Pyrrolidinone molecules break their hydrogen bonds from the one end of the long chain, forming a doubly hydrogen-bonded dimer. This hydrogen bond formation and breaking is a bidirectional process, stabilized by temperature.

When examining the diagonal of the square pattern (S_3_), it becomes evident that the NH band from longer molecular chains and DHBDs is involved in the processes of hydrogen bond breaking and formation. In contrast, shorter molecular chains remain inactive in these processes. Conversely, in shorter molecular chains, the NH bond exhibits coupling with CH vibrations, whereas in longer molecular chains and DHBDs, no significant coupling between NH and CH vibrational bands is observed. For instance, in the upper left quadrant of the spectra (CHNH coupling region), a weak but broad positive peak is observed. This peak represents a combination of two cross peaks: one arises from coherent coupling between the NH and the equatorial CH stretch vibration (ω_τ_ = 2890 cm^–1^, ω_*m*_ = 3300 cm^–1^), and the other originates from coherent coupling between the NH and the axial CH stretch vibration (ω_τ_ = 2953 cm^–1^, ω_*m*_ = 3280 cm^–1^). When square patterns are drawn to include the off-diagonal and axial/equatorial CH diagonal peaks, the upper right corners of both squares (S_4_ and S_5_) intersect with the diagonal NH peak at higher frequencies, corresponding to the short-chain SHBO. The stronger CHNH coupling observed in short chains compared to long chains can be explained as follows: In long chains, most NH groups are engaged in hydrogen bonding, which restricts their vibrational motion. As a result, the influence of NH vibrations on the neighboring axial or equatorial CH vibrations is reduced. In contrast, short chains form weaker hydrogen bonds, allowing greater freedom for NH vibrations, which in turn have a more pronounced effect on adjacent CH bands. Just below these positive coupling peaks, there is a negative peak at around (ω_τ_ = 2890 cm^–1^, ω_*m*_ = 3185 cm^–1^). This negative peak is a combination of two peaks, resulting from excited state absorption of the axial and equatorial CH bands, which are coupled with the NH band of SHBO.

It is expected that two positive peaks (opposite corner of the square pattern) would appear in the NHCH coupling region due to coherent coupling between the NH and axial/equatorial CH bands. However, these peaks are obscured by a strong, broad positive peak (to be discussed later) at (ω_τ_ = 3250 cm^–1^, ω_*m*_ = 2980 cm^–1^). This peak seems to appear due to a combination of two processes: (i) coherent coupling between the NH and CH vibrational bands and (ii) incoherent coupling between NH and CH vibrational bands. As shown in Figure 5b, the NH vibrational band lies at a higher energy than the first excited state of the CH vibrations. The NH vibrational band relaxes its energy into the CH band, which consequently promotes the CH vibration to its second excited state. Unlike coherent coupling, where participating oscillators exchange energy in a synchronized manner, incoherent coupling involves energy transfer from one oscillator to another without phase correlation or reciprocal exchange. The representative square pattern (yellow dotted square S_6_) suggests that short-chain oligomers are more likely involved in this coupling process. Additionally, the CH band associated with the C_3_ carbon atom (see Figure 1) appears to participate in the energy transfer.^31^ Since the NH vibrational band has higher energy than the CH band (see Figure 5b), energy flows only from the NH to the CH band, but not in the reverse direction. Consequently, no incoherent coupling peak opposite to this peak is observed in the CHNH coupling region.

**Figure 5:**
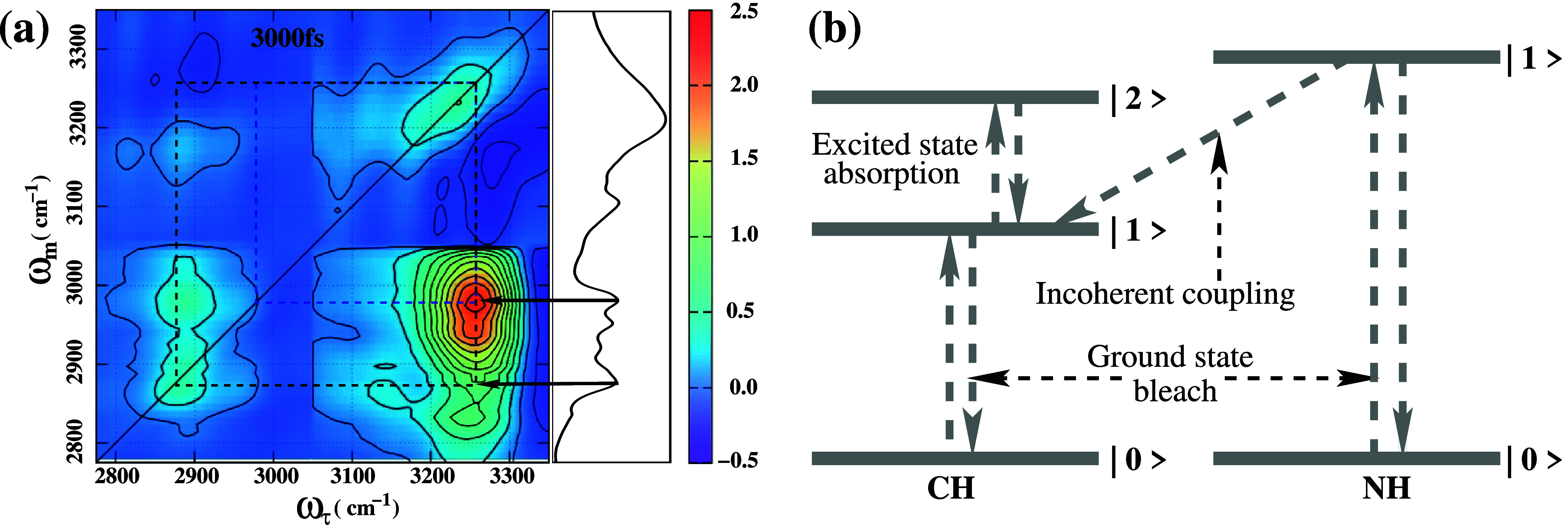
(a) 2DIR spectrum of 2-pyrrolidinone at waiting times of 3 ps. The peak intensities are scaled relative to the highest intensity peak shown in Figure 3. (b) Energy relaxation from the first excited state of the NH stretch vibration to the first excited state of the CH stretch vibration gives rise to the incoherent coupling peak in the 2DIR spectra.

The vibrational echo broadband spectrum at a population time *T*_*w*_ = 400 fs is shown in Figure 4a. All diagonal peaks appear with reduced intensity compared to those at *T*_*w*_ = 200 fs. Due to the short vibrational lifetime (∼1.5 ps) of the NH vibrational band^56,57^ the diagonal peak associated with NH band decays faster than the other peaks. This peak is also less broadened at the longer waiting time. In the CH vibrational region, the symmetric CH stretching vibrational peak on the diagonal decays slowly due to its longer lifetime,^58,59^ while the antisymmetric CH stretch peak decays faster than the symmetric one.

In the upper left corner of the spectra, the CHNH coherent coupling peak (ω_τ_ = 2890 cm^–1^, ω_*m*_ = 3300 cm^–1^) becomes more prominent. This peak shows a slightly elongated shape perpendicular to the diagonal. A corresponding negative peak appears at ω_τ_ = 2890 cm^–1^, ω_*m*_ = 3185 cm^–1^. These peaks are more intense at *T*_*w*_ = 400 fs compared to *T*_*w*_ = 200 fs, as the spectrum is normalized to the rapidly decaying NH stretch band. The presence of positive and negative peaks in the CHNH coupling region is characteristic of coherent coupling, where coherence is transferred from the CH stretch vibration to the NH stretch vibration.^60^

No new features appear in the lower right quadrant, which continues to be dominated by a single positive peak, though its intensity is reduced compared to *T*_*w*_ = 200 fs. This peak is attributed to incoherent coupling between the NH and CH stretch vibrations. The amplitude of this incoherent coupling peak remains significant relative to the coherent coupling peaks, reflecting the strong vibrational interaction between the NH and CH stretches. Consequently, the coherent coupling peaks are still overshadowed by the dominant incoherent coupling feature.

Figure 4b shows the broadband echo spectra at the next population time, *T*_*w*_ = 800 fs. The intensity of the diagonal peak in the NH vibrational region has now decreased to nearly half of its level at *T*_*w*_ = 400 fs. The excited state absorption peak at (ω_τ_ = 3310 cm^–1^, ω_*m*_ = 3180 cm^–1^) also shows reduced intensity. With its longer lifetime, the symmetric CH vibrational mode decays slowly and remains prominent along the diagonal, while the antisymmetric CH vibrational mode fades significantly. Both diagonal peaks in the CH vibrational region appear less elongated, reflecting decreased inhomogeneous broadening with increased waiting time. A distinct positive off-diagonal peak at ω_τ_ = 2890 cm^–1^, ω_*m*_ = 2980 cm^–1^ strongly suggests coupling between the symmetric and antisymmetric CH vibrational modes. The intensifying coupling peaks indicate chemical exchange, which interconverts symmetric and antisymmetric stretch vibrations.^49^ This process may be attributed to a pseudorotational bending that converts axial into equatorial CH bonds (see Figure 1). Additionally, the cross peaks in the CHNH coupling region are now more pronounced, with both positive and negative peaks showing elongation perpendicular to the diagonal.

The positive cross peak in the NHCH coupling region loses its intensity significantly and appears less elongated than before. The different modes show anticorrelation due to varying coupling constants, likely as a result of chemical exchange. This elongation originates from chemical exchange, where one conformer converts into another, altering the coupling between the CH and NH vibrational modes and resulting in anticorrelated cross peaks. Additionally, a negative peak (ω_τ_ = 3250 cm^–1^, ω_*m*_ = 2900 cm^–1^) now appears in the NHCH coupling region at 800 fs, caused by the anharmonicity of the NHCH combination tone; at earlier times, this peak was obscured by the positive peak.

Figure 4c presents the broadband echo spectrum at a population time of *T*_*w*_ = 1500 fs. In the NH vibrational region, the diagonal peak shows further attenuation, while in the CH vibrational region, the diagonal peaks remain distinctly visible due to the long lifetime of the CH band, although less intense compared to earlier population times. The coupling between the symmetric and antisymmetric peaks becomes more prominent, supporting the hypothesis that chemical exchange is an underlying process.

The coherent coupling peaks in the CHNH coupling region remain highly prominent. While they experience some loss in intensity, they become more pronounced as diagonal peaks diminish more rapidly. The elongation of these positive and negative off-diagonal peaks, oriented perpendicular to the diagonal, remains evident in the spectrum.

The cross peak in the NHCH coupling region, originating from the incoherent coupling between the NH stretching mode and the antisymmetric CH stretching mode, is no longer present in the spectrum. This incoherent coupling peak, resulting from energy transfer from the NH and CH stretching bands, appears to vanish at *T*_*w*_ = 1500 fs. As a result, the coherent coupling peaks at (ω_τ_ = 3210 cm^–1^, ω_*m*_ = 2890 cm^–1^) and (ω_τ_ = 3210 cm^–1^, ω_*m*_ = 2990 cm^–1^), previously obscured, now become distinctly visible.

The broadband spectrum at the next higher population time, *T*_*w*_ = 3000 fs, is shown in Figure 5. The intensities of all diagonal peaks decrease significantly. A particularly intriguing cross peak appears in the NHCH coupling region. The cross peak at ω_τ_ = 3250 cm^–1^, ω_*m*_ = 2980 cm^–1^, attributed to the incoherent coupling between the NH and CH stretching modes at early population times, had gradually weakened and disappeared by *T*_*w*_ = 1500 fs. However, it re-emerges strongly at the same position at *T*_*w*_ = 3000 fs. This behavior is attributed to the chemical exchange of the CH mode in the 2-pyrrolidinone molecule, transitioning from the equatorial configuration to the axial configuration and then back to the equatorial configuration (see Figure 1). At early population times, the population of axial configuration species remain higher than equatorial species, enabling significant energy transfer from the higher-energy NH stretch vibrational mode to the lower-energy CH stretch vibrational mode via incoherent coupling (see Figure 5b). As the population time increases, the molecule undergoes structural rearrangement toward the equatorial configuration. This rearrangement increases the spatial separation between the NH and CH bands, reducing energy transfer between them. At approximately 1500 fs, the populations of the axial and equatorial species reverse, and as a result, no significant energy transfer occurs between the NH and CH vibrational bands. By *T*_*w*_ = 3000 fs, the molecule returns to its initial population of axial species, causing the NHCH coupling cross peak to reappear with nearly the same intensity as at earlier times. As the lifetimes of all characteristic peaks are less than or approximately 3 ps, most peaks decay and disappear, leaving only a few visible at very low intensity.

As noted in the above discussion, the diagonal and off-diagonal peaks exhibit distinct time evolution. As examples, the time-dependent behavior of three representative chemical processes is shown in Figure 6. The diagonal peak at ω_τ_ = ω_*m*_ = 3225 cm^–1^ exhibits an exponential decay with population time (*T*_*w*_), as shown in Figure 6a, which is characteristic of diagonal peaks. The calculated lifetime of the NH vibration is 1.2 ps, in good agreement with previously reported values.^57^ Figure 6b depicts the evolution of the cross peak at (ω_τ_ = 2862 cm^–1^, ω_*m*_ = 2950 cm^–1^), arising from the chemical exchange between the axial and equatorial CH bands. This cross peak initially grows rapidly with waiting time and eventually reaches saturation at around 3 ps, which is consistent with the lifetime of the axial and equatorial CH vibrations.^61^

**Figure 6:**
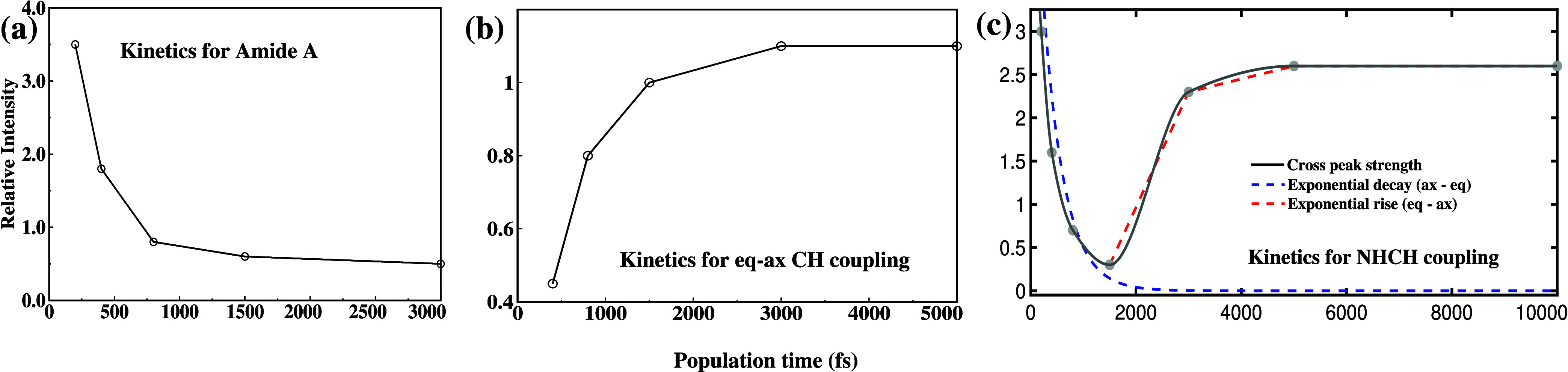
Time evolution of three representative chemical processes: (a) Evolution of diagonal peak at the NH vibrational band. (b) Evolution of coherent coupling peak between axial and equatorial CH vibrational bands. (c) Kinetics of incoherent coupling peak between NH and CH vibrational bands.

The time evolution of the NHCH coupling peak at (ω_τ_ = 3250 cm^–1^, ω_*m*_ = 2980 cm^–1^) is presented in Figure 6c. The black continuous curve represents the overall evolution of the peak, while the red and blue dotted lines decompose the process into its components. The blue curve illustrates the exponential decay corresponding to energy flow from the NH to the CH vibrational band, whereas the red curve represents the chemical exchange between the axial and equatorial configurations of the 2-pyrrolidinone molecule. The red curve reaches saturation after 3 ps.

## Conclusions

This article presents a study on the ultrafast vibrational dynamics of 2-pyrrolidinone using broadband two-dimensional infrared (2DIR) spectroscopy. The spectral range includes the CH and NH vibrational bands, enabling the investigation of various dynamical processes among these vibrations. The role of intermolecular hydrogen bonding, which significantly impacts the molecular structure and dynamics of 2-pyrrolidinone, is examined in detail.

At room temperature, 2-pyrrolidinone forms doubly hydrogen-bonded dimers (DHBDs) and single hydrogen-bonded oligomers (SHBOs) of varying sizes. The study reveals that DHBDs and long-chain SHBOs actively participate in hydrogen bond breaking and reformation processes, whereas short-chain SHBOs remain largely inactive in these dynamics. Given the critical role of hydrogen bonding in biological processes, these findings provide valuable insights into biological activities.

Additionally, the presence of cross peaks in the CHNH region confirms coupling between the NH vibrational band and various CH vibrational bands. Such coupling has not been previously reported, primarily due to the unavailability of broadband lasers with sufficient energy. A strong positive peak in the NHCH coupling region suggests energy relaxation from the NH to CH vibrational modes. The exponential decay of this peak’s intensity at early times, followed by its reappearance at longer waiting times, indicates molecular configurational changes, where the molecule transitions from axial to equatorial configurations and then returns to the axial state.

Overall, the spectra discussed here provide comprehensive structural and dynamical information about 2-pyrrolidinone, offering valuable insights into its behavior and its potential implications for understanding biological processes.
